# Australia could miss the WHO hepatitis C virus elimination targets due to declining treatment uptake and ongoing burden of advanced liver disease complications

**DOI:** 10.1371/journal.pone.0257369

**Published:** 2021-09-16

**Authors:** Jisoo A. Kwon, Gregory J. Dore, Behzad Hajarizadeh, Maryam Alavi, Heather Valerio, Jason Grebely, Rebecca Guy, Richard T. Gray

**Affiliations:** Kirby Institute, UNSW Sydney, Sydney, New South Wales, Australia; Centers for Disease Control and Prevention, UNITED STATES

## Abstract

Australia was one of the first countries to introduce government-funded unrestricted access to direct-acting antiviral (DAA) therapy, with 88,790 treated since March 2016. However, treatment uptake is declining which could potentially undermine Australia’s progress towards the WHO HCV elimination targets. Using mathematical modelling, we updated estimates for those living with chronic HCV in Australia, new cases of decompensated cirrhosis (DC), hepatocellular carcinoma (HCC), and liver-related mortality among the HCV-cured and viraemic populations from 2015 to 2030. We considered various DAA treatment scenarios incorporating annual treatment numbers to 2020, and subsequent uptake per year of 6,790 (pessimistic), 8,100 (intermediate), and 11,310 (optimistic). We incorporated the effects of excess alcohol consumption and reduction in progression to DC and HCC among cirrhosis-cured versus viraemic individuals. At the end of 2020, we estimated 117,810 Australians were living with chronic HCV. New cases per year of DC, HCC, and liver-related mortality among the HCV viraemic population decreased rapidly from 2015 (almost eliminated by 2030). In contrast, the growing population size of those cured with advanced liver disease meant DC, HCC, and liver-related mortality declined slowly. The estimated reduction in liver-related mortality from 2015 to 2030 in the combined HCV viraemic and cured population is 25% in the intermediate scenario. With declining HCV treatment uptake and ongoing individual-level risk of advanced liver disease complications, including among cirrhosis-cured individuals, Australia is unlikely to achieve all WHO HCV elimination targets by 2030.

## Introduction

Chronic hepatitis C virus (HCV) infection is a global public health threat with 58 million people living with HCV [1], and an estimated 0.29 million liver-related deaths each year, from liver failure and liver cancer [1]. In 2015, the World Health Organization (WHO) set global targets to eliminate HCV as a major global public health threat by 2030 [2], including three specific targets: 80% of people with HCV treated, and declines in new HCV infections by 90%, and liver-related mortality by 65%. Australia was one of the first countries to introduce government-funded unrestricted access to direct-acting antiviral (DAA) therapy [3], with around 88,790 (estimated 45% of chronic HCV population) treated since March 2016. Treatment numbers have progressively declined, however, making updated projections of HCV elimination progress essential.

We have previously modelled the Australian HCV epidemic and estimated that all three WHO HCV elimination targets could be met if 14,000 people with HCV are treated each year from 2019, including liver-related mortality among HCV viraemic cases [4]. However, only 11,310 and 8,100 people were treated during 2019 and 2020, respectively, continuing a declining trend since 2016. This trend may not be associated with the COVID-19 pandemic and hence a return to higher treatment levels may not occur. In addition, cured patients, particularly those with cirrhosis, continue to have an elevated risk of liver-related morbidity and mortality, albeit at a lower risk than HCV viraemic individuals [5–7].

We update our modelling estimates annually to track HCV infection and resulting liver disease burden using new surveillance, clinical and treatment data [8]. In particular, estimates for the level of duplicate HCV notifications [9] and a revised estimate for spontaneous clearance [10] have been incorporated, both of which have resulted in a lower estimate of people living with chronic HCV in Australia and resulting liver disease burden. Therefore, here we provide updated estimates for the prevalence of HCV in Australia to the end of 2020 and discuss whether Australia can still meet the WHO elimination targets by 2030 with the current treatment uptake and the ongoing burden of advanced liver disease complications due to HCV infection, despite the rapid treatment uptake in 2016. We also estimated treatment rates required post-2021 for Australia to meet the WHO HCV elimination targets and determined the number of new cases of decompensated cirrhosis (DC), hepatocellular carcinoma (HCC), and liver-related mortality among the HCV viraemic *and* cured populations in Australia from 2015 to 2030.

## Materials and methods

The model we use for the Australian HCV estimates is based on the Bright model developed by the Center for Disease Analysis (CDA). This model was adapted to reflect Australian settings from a previous model [4] and is updated annually to include data to the end of each year for HCV prevalence, number of diagnoses, and DAA uptake and to capture specific characteristics of Australia’s HCV epidemic.

### Model structure

**Fig 1:**
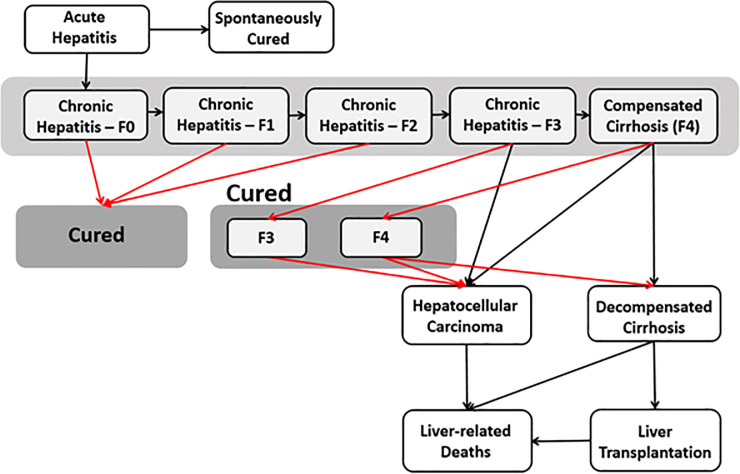
HCV model schematic diagram showing disease progression (black lines: Viraemic, red lines: Cured).

The model has been described in detail previously [4, 11]. Here we provide a summary focusing on the key features of relevance for this update and analysis. The model is a compartmental difference equation model implemented in Microsoft Excel (Redmond, WA) with the overall Australian population categorized by age, sex, and HCV disease stage (as shown in Fig 1). As described previously, we assume HCV cure following DAA therapy reduces the liver-related mortality rate among people living with DC and HCC by 50%. This is based on recent evidence from HCV linkage studies in NSW, Australia which showed a two to three-fold increase in HCC survival following treatment in the DAA era [12]. The disease progression rate among the cured population is lower compared to the viraemic population (Table 1).

**Table 1:** Model inputs and parameter estimates.

Parameters				Value						Reference	
**Population size and age distribution (1990–2030)**							[4]
**Total viraemic cases (2015)**				188,690			Revised calculation as described in the main text
**Newly HCV diagnoses (2020)**				8,110			[20]
**Number of people treated**				See below			Updated from [21, 22]
**Disease progression rates**							
**Acute to F0**				4.4% - 21.8%			[23, 24]
**F0 to F1**				3.4%– 14.3%			[23, 24]
**F1 to F2**				4.5%– 22.4%			[23, 24]
**F2 to F3**				4.7%– 20.0%			[23, 24]
**F3 to F4**				0.24%			[25]
**F3 to HCC**				3.0%			[25]
**F4 to DC**				3.6%			[7, 25]
**F4 to HCC**				20.0%			[5, 24, 26, 27]
**DC liver-related mortality**				70.7%			[24, 26, 27]
**HCC liver-related mortality**				16.2%			[24, 26, 27]
**1** ^**st**^ **year**				4.4% - 21.8%			[23, 24]
**Subsequent years**				3.4%– 14.3%			[23, 24]
**Reduction in progression rate from F3 to HCC (cured)**				77%			[5]
**Reduction in progression rate from F4 to DC (cured)**				76%			[6, 7]
**Reduction in progression rate from F4 to HCC (cured)**				77%			[5]
**Reduction in liver-related mortality among cured DC and HCC population**				50%			[12]
**Relative risk of having cirrhosis among population with excessive alcohol consumption (>50g per day)**				1.7–3.3			[15, 16]
**Spontaneously clearance rate**				28%			[10]
**Background mortality rate**				1.76%			[28]
**SVR rate (F0-F3)**				95%			[29–32]
**SVR rate (F4)**				90%			
	**Treatment roll-out scenarios**						
	**2015 (interferon + DAA)**	**2016**	**2017**	**2018**	**2019**	**2020**	**Post-2021**
**Pessimistic roll-out**	4,720	33,200	20,970	15,210	11,310	8,100	6,790
**Intermediate roll-out**	4,720	33,200	20,970	15,210	11,310	8,100	8,100
**Optimistic roll-out**	4,720	33,200	20,970	15,210	11,310	8,100	11,310

Abbreviations: F0–F4 = Fibrosis stage from F0 to F4; DC = decompensated cirrhosis; HCC = Hepatocellular carcinoma; SVR = Sustained virological response. Note: Pessimistic roll-out: 40% decrease of DAA uptake from 2018; Intermediate roll-out: DAA uptake remains at 2019 level; Optimistic roll-out: Where DAA uptake remains at 2018 level.

The model incorporated interferon-based (IFN-based) therapy, prior to the introduction of DAA therapy, with SVR rates depending on regimen and genotype [4, 13, 14]. From 2016, we assumed SVR rates for DAA therapy from clinical studies including disease stage at initiation (Table 1).

### Updated estimate of 2015 base prevalence

The model uses the estimated HCV prevalence at the end of 2015 as a calibration target for the model and as a starting point for treatment scenarios. Previously we estimated 227,000 people were living with chronic HCV in Australia at the end of 2015 [4]. We have updated the model to reflect recently available data and to incorporate improved knowledge of the roll-out and impact of DAA therapy on HCV epidemics. To estimate the number of people living with HCV in 2015 we used cumulative notifications and spontaneous clearance, mortality, and migration rate estimates. Recent linkage studies being conducted in the Australian states of New South Wales (NSW) and Victoria estimated that 7 to 11% of notifications were duplicates [9]. Given this evidence and the level of uncertainty in duplication, we assumed 9% (range 7–11%) of all notifications are duplicates. Furthermore, recent evidence from a linked cohort in British Columbia [10] suggests the proportion of HCV diagnoses who were HCV RNA negative is 28% (3% higher than what we used previously). These adjustments combined produce a 12% reduction in the cumulative HCV notifications and a lower overall estimate for the number of people living with HCV in Australia at the end of 2015.

### Updated estimates of morbidity and mortality due to HCV infection

To improve our estimates of liver-related mortality in the model, we incorporated a higher risk of developing cirrhosis, DC, HCC, and liver-related mortality among the population with excessive alcohol consumption (defined as > 50g per day). For example, people living with HCV who have excessive alcohol consumption are 2.3 (95% CI 1.7–3.3) times more likely to develop cirrhosis [15, 16]. We used available progression rates for those with and without excessive alcohol consumption from published literature and the percent of HCV infected people with DC or HCC that have excess alcohol consumption based on hospital admissions for alcohol use disorder from the NSW, Australia linkage study. The study showed the percentage of patients with alcohol use disorder among HCV infected people with DC and HCC increased from 14% to 51% over 2001–2018. We adjusted the percentage of people with alcohol use disorder who do not have DC or HCC in our updated model to match the number of liver-related deaths from the NSW linkage study. This resulted in an estimate of 19% with excessive alcohol consumption who do not have DC or HCC, which aligns with available data from the separate NSW linkage and Enhancing Treatment of Hepatitis C in Opioid Substitution Settings (ETHOS) studies [17].

### Updated DAA treatment data and projection scenarios

The proportion of DAA treatments initiated by patients in each fibrosis stage was estimated using the Real-world Efficacy of Antiviral therapy for Chronic Hepatitis C (REACH-C) [18] and Pharmaceutical Benefits Scheme (PBS) data [19]. From March 2016-to the end of 2020, 88,790 people living with chronic HCV received DAA therapy. Of those, 23–27% were in F0, 23–27% were in F1, 15–17% were in F2, 10% were in stage F3, 20–30% were in stage F4. During 2016–2020, the proportion who received treatment in each disease stage was relatively stable. We assumed treatment disease stage distribution post-2021 remained at the 2020 level with recent evidence of stable treatment uptake in each disease stage throughout 2016–2020.

As in our previous work, we developed three treatment scenarios from the end of 2020 based on the estimated number of DAA therapy initiations from 2016 to 2020. Post-2021 we assumed a pessimistic roll-out corresponding to 40% less people being treated with DAA therapy than for 2019, an intermediate roll-out corresponding to the annual number treated equaling the number in 2020, and an optimistic scenario where the annual number treated increased back to the 2019 level. Table 2 shows the corresponding treatment numbers for Australia. For comparison purposes, we also ran an IFN-based (pre-DAA listing) scenario where the number treated remains at the 2015 level (n = 4,720) and new HCV infections are held constant until 2020.

**Table 2:** The key HCV estimates with treatment roll-out scenarios (best estimate, 95% CI^†^).

	End of 2015	2030		
	Baseline (2015)	Pessimistic treatment scenario	Intermediate treatment scenario	Optimistic treatment scenario
**People living with chronic HCV**	188,690	60,370	48,140	19,290
	(170,620–203,530)	(35,500–81,760)	(24,000–69,650)	(4,860–44,630)
**New infections (all)**	5,600	1,310	1,010	280
	(5,350–6,110)	(610–2,080)	(210–1,860)	(40–1,300)
**Chronic HCV prevalence**	0.8%	0.2%	0.2%	0.1%
	(0.7%-0.9%)	(0.1%-0.3%)	(0.1%-0.3%)	(0.0%-0.2%)
**Fibrosis** ^‡^				
**F0**	52,600	8,610	6,030	750
	(40,430–70410)	(2,100–20,040)	(560–17,410)	(130–10,940)
**F1**	64,670	16,160	12,910	5,080
	(55,650–72,300)	(5,760–30,050)	(2,690–26,780)	(50–18,760)
**F2**	28,100	9,150	7,280	2,680
	(25,120–30,480)	(3,630–14,400)	(1,790–12,510)	(8–7,870)
**F3**	25,840	15,760	13,800	8,990
	(15,000–37,590)	(7,270–24,070)	(5,660–21,760)	(1,970–16,160)
**F4**	14,970	9,280	7,090	1,630
	(8,650–23,170)	(1,710–16,100)	(1,170–13,770)	(480–8,080)
**Decompensated cirrhosis**	1,300	650	430	40
	(640–2,560)	(20–1,760)	(4–1,470)	(3–820)
**HCC** ^§^	670	430	340	100
	(370–1,320)	(50–1,060)	(30–930)	(10–610)
**New Decompensated cirrhosis cases**				
**Viraemic**	420	250	180	20
	(250–680)	(20–480)	(4–410)	(2–230)
**Viraemic + cured**	430	410	360	230
	(260–700)	(140–680)	(130–620)	(130–480)
**New HCC cases**				
**Viraemic**	540	330	240	40
	(310–870)	(50–590)	(20–510)	(8–300)
**Viraemic + cured**	550	520	460	290
	(310–880)	(200–840)	(180–770)	(160–610)
**Annual liver-related mortality**				
**Viraemic**	730	420	310	50
	(410–1,200)	(40–850)	(20–720)	(10–440)
**Viraemic + cured**	740	650	550	330
	(410–1,220)	(210–1,140)	(200–1,030)	(180–790)
**Cumulative cases over 2016–2030**	**Baseline**	**Pessimistic**	**Intermediate**	**Optimistic**
**New infections (all)**	73,420	48,650	47,110	43,330
	(68,930–82,540)	(41,340–58,180)	(39,310–56,990)	(35,700–54,080)
**New Decompensated cirrhosis cases**				
**Viraemic**	8,320	3,030	2,670	1,800
	(4,650–13,410)	(480–6,430)	(380–6,050)	(370–5,160)
**Viraemic + cured**	9,040	4,830	4,540	3,820
	(5,200–14,310)	(1,740–8,750)	(1,670–8,450)	(1,730–7,750)
**New HCC cases**				
**Viraemic**	10,730	4,220	3,780	2,700
	(5,740–16,700)	(960–8,290)	(840–7,830)	(720–6,700)
**Viraemic + cured**	11,610	6,380	6,020	5,130
	(6,430–17,800)	(2,510–11,090)	(2,430–10,710)	(2,320–9,830)
**Annual liver-related mortality**				
**Viraemic**	15,170	5,880	5,310	3,980
	(7,850–24,420)	(1,340–12,530)	(1,230–11,910)	(1,160–10,420)
**Viraemic + cured**	16,020	7,930	7,430	6,260
	(8,510–25,480)	(2,840–15,110)	(2,750–14,570)	(2,670–13,290)
**Years of achieving WHO HCV elimination targets**				
		**Pessimistic**	**Intermediate**	**Optimistic**
90% reduction in new chronic infections		2034	2032	2030
80% of people living with chronic HCV treated		2032	2030	2027
65% reduction in HCV-related mortality				
Viraemic only		2039	2033	2023
Viraemic and cured		>2050	>2050	>2050

Note

^†^CI (confidence interval)

^‡^Fibrosis stage from 0 to 3

^§^Hepatocellular carcinoma (HCC)

### Calculation of treatment coverage

To calculate treatment coverage on an ongoing basis for tracking towards HCV elimination targets, we considered new infections, cured people, and mortality during the DAA era. The treatment coverage for HCV in a given year was defined as the number of people who have been treated and are still living since the end of 2015 at the end of the year divided by the number of people who have lived with chronic HCV since the end of 2015 (including still living with HCV and those cured) at the end of the previous year.

### Model calibration

The detailed methodology used for calibration has been described previously [4]. With the updated notification and treatment data we estimated the 2015 base prevalence at 188,690 people. The model was calibrated to this prevalence and 8,110 new diagnoses at the end of 2020 (with duplicates removed; Table 1, additional model inputs are in S1 Table in S1 File) by calculating the annual HCV incidence using a constant multiplier describing the relative incidence compared to the incidence in 1950.

### Generation of results

The Oracle Crystal Ball add-in (Oracle Corp) for Excel software was used to perform 1000 Monte Carlo simulations (using samples from the parameter ranges in Table 1) and estimate 95% confidence intervals (CIs) for the model outputs up to 2030 for each treatment scenario. The final spreadsheet used to produce all the results is available upon reasonable request.

This modelling study used publicly available and reported surveillance data and did not require ethics approval. All data are in an aggregated and de-identified format.

## Results

At the end of 2015, an estimated 188,690 people were living with chronic HCV. Of those, 81% were diagnosed with HCV and 22% (n = 33,200) of those diagnosed received treatment during 2016. Among people living with chronic HCV in 2015, 28% were in stage F0, 34% were in stage F1, 15% were in stage F2, 14% were in stage F3, and 9% were in stage F4 (cirrhosis) (Table 2). In 2015, an estimated 740 (95% CI: 410–1,220) people living with chronic HCV died with HCV-related liver disease.

At end 2020, an estimated 117,810 (95% CI: 96,510–135,640) people were living with chronic HCV. Of those, 77% (n = 90,560; 95% CI: 57,010–131,500)) were diagnosed with HCV and 8% (n = 8,100) of people living with chronic HCV and diagnosed at end 2019 (n = 95,870) received treatment in 2020. Around 88,790 people living with chronic HCV in Australia received DAA therapy during 2016–2020, equating to approximately 45% treatment coverage by the end of 2020. Among people living with chronic HCV at end 2020, 24% were in stage F0, 35% were in stage F1, 16% were in stage F2, 18% were in stage F3, and 7% were in stage F4 (cirrhosis). Among people living with chronic HCV at end 2019 and received treatment during 2020, uptake varied by disease stage: 7% (n = 2,150) of people in stage F0, 5% (n = 2,150) in F1, 7% (n = 1,380) in F2, 4% (n = 810) in F3, and 23% (n = 1,480) in F4.

### Reductions in HCV prevalence, incidence, and liver-related mortality due to DAA

**Fig 2:**
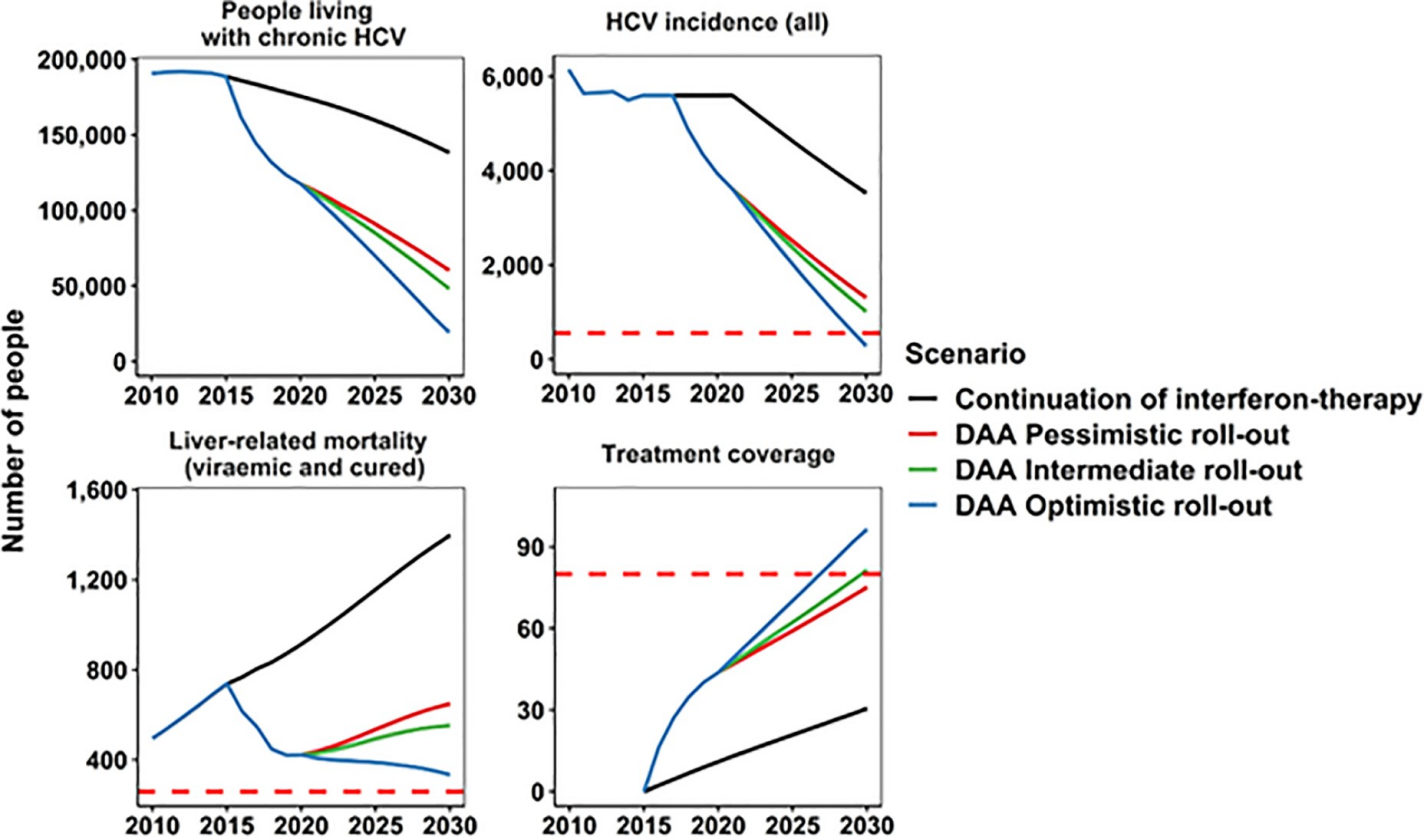
Annual change in people living with chronic HCV, HCV incidence, treatment coverage, and liver-related mortality in Australia 2010–2030 compared to WHO HCV elimination targets (red dotted lines: 90% reduction in incidence, 80% eligible treated, 65% reduction in deaths).

**Fig 3:**
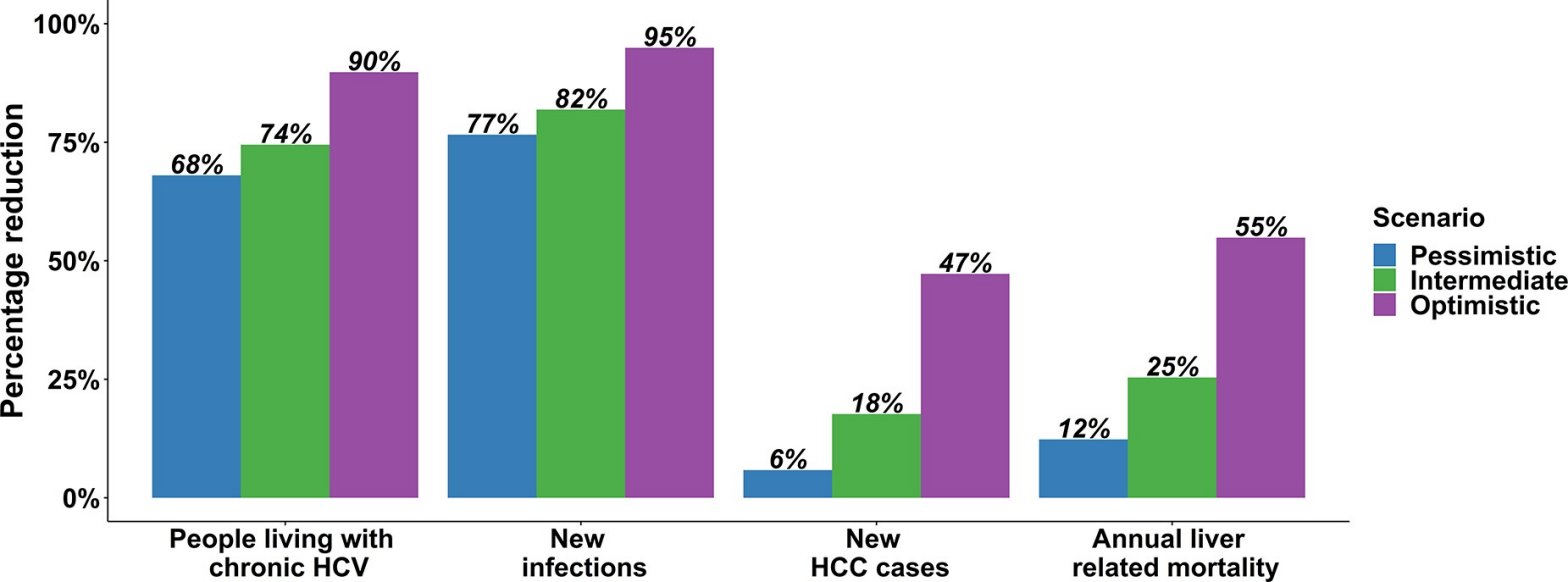
Percentage reductions in PLHCV, new infections, new HCC cases (viraemic and cured), liver-related mortality (viraemic and cured), in 2030 compared to 2015 (blue bar: Pessimistic scenario, green bar: Intermediate scenario, purple bar: Optimistic scenario).

In Australia, there were substantial reductions in chronic HCV prevalence, new HCV cases per year (incidence), and liver-related mortality in all DAA treatment scenarios (Fig 2). The chronic HCV prevalence decreased to 60,370 (95% CI: 35,000–81,760; 68% reduction) by 2030 (Table 2, Fig 3) in the pessimistic scenario, and to 48,140 (95% CI: 24,000–69,650; 74% reduction) and 19,290 (95% CI: 4,860–44,630; 90% reduction) in intermediate and optimistic scenarios in 2030, respectively. Compared to the incidence in 2015, the HCV incidence in 2030 was 77%, 82%, and 95% lower in the pessimistic, intermediate, and optimistic scenarios, respectively (Fig 3).

**Fig 4:**
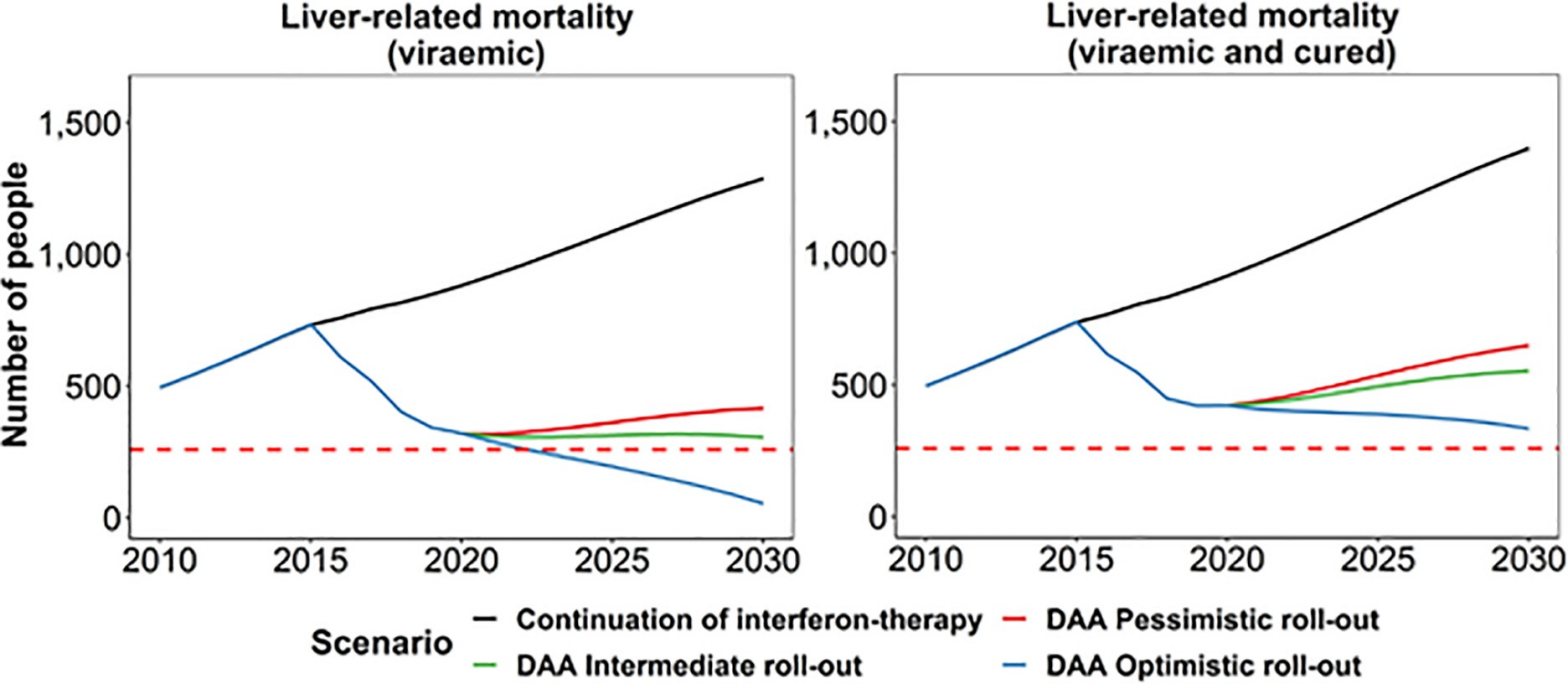
Liver-related mortality among viraemic only and viraemic and cured combined in Australia 2010–2030 compared to WHO HCV mortality target (red dotted lines: 65% reduction from 2015).

The new HCV cases per year (incidence) of DC, HCC and liver-related mortality among the HCV viraemic population decreased rapidly with DAA therapy scale-up due to the reduction in those remaining viraemic with advanced fibrosis (with liver-related mortality almost eliminated by 2030). In contrast, despite the reduced risk or progression among those with cirrhosis who were cured, the growing population size of people with advanced fibrosis among the cured population meant that DC, HC, and liver-related mortality declined slowly. In the intermediate scenario, there remained an estimated 180 (95% CI: 130–220) new cases of DC, 210 (95% CI: 160–260) new cases of HCC, and 250 (95% CI: 180–310) liver-related mortality among the cured population in 2030. Among the combined viraemic and cured population, the estimated number of liver-related deaths was 650 (95% CI: 210–1,140), 550 (95% CI: 200–1,030), and 330 (95% CI: 180–790) in the pessimistic, intermediate, and optimistic scenarios in 2030, respectively (Fig 2). The estimated liver-related mortality among the combined viraemic and cured population in 2030 is 12%, 25%, and 55% lower compared to 2015, in the pessimistic, intermediate, and optimistic scenarios, respectively (Fig 3). In contrast, the estimated liver-related mortality among the viraemic population in 2030 is 43%, 58%, and 93% lower compared to 2015, in the pessimistic, intermediate, and optimistic scenarios, respectively (Fig 4).

In the intermediate scenario, the model projected that Australia can achieve the WHO HCV elimination incidence and treatment targets by 2032 if the current treatment uptake remains the same (90% reduction in incidence in 2032, and 80% treated in 2030, see Fig 2 and Table 2). The pessimistic scenario will meet the WHO incidence and treatment targets by 2034 (90% reduction in incidence in 2034, and 80% treated in 2032), and the optimistic scenario will meet the WHO incidence and treatment targets by 2030 (90% reduction in incidence in 2030, and 80% treated in 2027). The 65% reduction of liver-related mortality target is only achievable with the optimistic scenario in 2023 when only the viremic population is considered but difficult to achieve when viraemic and cured liver-related deaths are combined (Table 2, Fig 2).

Our sensitivity analysis showed that the year of meeting the WHO HCV mortality target is sensitive to the risk of developing cirrhosis among people living with chronic HCV who have excessive alcohol consumption. The year of meeting the WHO mortality target ranged from 2042 to after 2050 in the intermediate scenario. Our analysis showed that Australia needs to treat at least 16,790 (post-2021, 107% higher than 2020 level) people living with chronic HCV per year to meet the WHO mortality target by 2030. We also ran the model without excess alcohol consumption which showed that the year the WHO mortality target among viraemic cases is met in 2021 in the intermediate scenario but this target would not be achieved if viraemic and cured cases are considered (S2 Table in S1 File).

## Discussion

With the updated mathematical model incorporating complete national datasets on HCV diagnoses and treatment as well as effects of alcohol use disorder, we found that Australia cannot meet the WHO HCV incidence and treatment targets by 2030 if the current treatment uptake remains at the 2020 level. The 28% reduction in treatment uptake in 2020 compared to 2019 is consistent with the decreasing trend in treatment and testing in Australia [33]. Our analysis highlights that Australia needs to treat at least 16,790 (post-2021, 107% higher than 2020 level) people living with chronic HCV per year to meet all WHO targets by 2030.

Our study showed that the ability to meet the 65% reduction in HCV liver-related mortality target depends on how mortality is defined. This is consistent with our previous findings [4] but we have updated the model to include other risk factors that significantly impact mortality post-cure, such as an increased risk of developing cirrhosis among people who have excessive alcohol consumption. We also updated the model to include new data on the proportion of people living with chronic HCV who initiated DAA treatment in each fibrosis stage obtained from the REACH-C study and the Pharmaceutical Benefits Scheme. When HCV liver-related mortality includes people with chronic HCV and those who are cured, the target will be difficult to be met by 2030 even under the optimistic scenario as the increasingly large cured population continues to contribute to HCV-related deaths. Australia is one of the leading countries for the roll-out of DAA therapies providing unrestricted access to people living with HCV, but we have demonstrated that Australia will still find it hard to achieve the WHO HCV mortality target. The increasing trajectory of liver-related mortality prior to the DAA era, due to the ageing chronic HCV population, is a key factor in determining the feasibility of mortality targets with 2015 as the baseline. When HCV liver-related mortality includes only people with chronic HCV, then a 65% reduction would only be achieved under an optimistic treatment scenario. This study, therefore, highlights a need to more clearly define the WHO HCV mortality indicator.

This is an updated study that builds on a previous model [4, 34] by incorporating the ongoing risk of DC and HCC among individuals who have been cured and increased risk among individuals with excessive alcohol consumption. The model used the most up-to-date national datasets, including the total number of people who are treated in 2016–2020, and the relatively higher treatment uptake among those in more advanced liver disease stages. In our model we implicitly assumed testing rates were sufficient for the appropriate number of people to be treated and that harm reduction programs remained at the 2016 level, to focus on the impact of treatment as prevention strategies. Another Australian modelling study by Scott et al. [35] suggested that the WHO incidence target of a 90% reduction would not be reached if the current testing rate remains the same. Our results also suggest that WHO HCV incidence and treatment coverage targets will no longer be met by 2030 under the intermediate scenario (annual number treated equaling the number in 2020). Both studies highlight the challenge of maintaining or reaching the high treatment rate required to achieve the WHO HCV elimination targets. Around 88,790 people living with chronic HCV in Australia received DAA therapy in 2016–2020, equating to approximately 45% treatment coverage by the end of 2020. The treatment coverage of 80% will be achieved by 2030 with the intermediate scenario and by 2027 with the optimistic scenario.

People living with DC and HCC remain at risk of liver disease and liver-related death. Even with the reduced risk of progression from advanced fibrosis to DC and HCC in those who are cured, many deaths among this relatively large population will continue to occur. The rate of mortality among people who develop advanced liver disease complications is particularly high, especially in those with a history of alcohol use disorder [36]. There are additional strategies that could further reduce mortality beyond the impact of DAA therapy. First, alcohol abstinence among those with advanced fibrosis, even if cured, is likely to reduce the risk of DC and HCC and thus liver-related mortality. Second, regular HCC screening among those with advanced fibrosis should increase early detection of HCC and the uptake of curative HCC management. Third, enhanced access to liver transplantation could provide further benefits. For example, people with ongoing alcohol or drug use have generally been de-prioritized for transplantation.

There are several limitations in this study. The model did not specifically include DAA uptake among at-risk populations such as people who inject drugs and prisoners. However, evidence suggests that DAA uptake among people who inject drugs has been even higher than the broader chronic HCV population (47% among people with drug dependence or current injecting compared to 38% among the broader population) [37, 38]. The prevalence of heavy alcohol consumption was based on hospital admissions for alcohol use disorder from an Australian HCV data linkage study. This might underestimate the true number of people with heavy alcohol consumption and is therefore conservative. The model also did not track the reinfection of people who cleared the virus. Instead, the population who cleared the virus will return to the susceptible pool and have the same risk of HCV reinfection as for primary HCV. Again, this should be conservative, as the incidence of HCV reinfection is generally lower than primary infection. In the previous study, we showed that the model is quite sensitive to the level of mortality reduction post-cure among those with DC and HCC. We used a 50% reduction from the linkage study of HBV patients and conducted a sensitivity analysis varying the mortality reduction from 0% to 80%. In the current study, we used results from ongoing linkage studies in Australia which showed a two to three-fold increase in HCC survival following treatment in the DAA era. Further iterations of HCV elimination progress and target feasibility will be required as DAA treatment is updated and further information is gathered on several model parameters.

There are several important clinical and policy implications of this study. This study has demonstrated that it will be very difficult to reach the WHO HCV elimination targets in Australia under the current treatment scenarios. This is particularly concerning given the reductions in HCV testing and treatment during the COVID-19 pandemic which will further delay the attainment of elimination targets [39]. As such, it will be important to consider the implementation of evidence-based interventions that have been shown to enhance HCV testing [40, 41], including medical chart reminders [40], dried-blood-spot testing [42], point-of-care HCV antibody testing [43–46], point-of-care HCV RNA testing [47–49], and reflex HCV RNA testing [50–52]. It will also be essential to implement strategies that have been demonstrated to increase treatment, including patient navigation or care coordination [53–55] and integration of HCV care into drug treatment settings [56, 57]. Scale-up of evidence-based interventions to enhance HCV testing and treatment will be critical to achieve the increases in treatment uptake required to meet the WHO viral hepatitis elimination targets. The information from this study is important for both national and international policies and viral hepatitis strategies to inform target setting and facilitate the development of feasible actions and workplans to achieve the WHO viral hepatitis elimination targets nationally and globally.

In conclusion, our analysis demonstrated that meeting WHO HCV elimination targets will be difficult to achieve if the current treatment uptake remains at the 2020 level. Our study highlights that treatment uptake level needs to be increased by 107% to meet all WHO targets by 2030. Furthermore, our study highlighted that even if a large population of people with viraemic HCV are cured there will still be high levels of liver disease, DC, HCC and liver-related mortality. The liver-related mortality targets by 2030 will be difficult to achieve if the mortality indicator includes people who are cured due to the ongoing risk of DC and HCC among this population, and increased risk of progression post-cure among those with alcohol use disorder.

## Supporting information

S1 File (DOCX)
