# Supplementary material for: Australia could miss the WHO hepatitis C virus elimination targets due to declining treatment uptake and ongoing burden of advanced liver disease complications
Source: PLoS One. 2021 Sep 16;16(9):e0257369. doi: 10.1371/journal.pone.0257369 (PMC8445464; doi:10.1371/journal.pone.0257369)
Supplement: S1 File — (DOCX) [file pone.0257369.s001.docx]

**Australia could miss the WHO hepatitis C virus elimination targets**

**due to declining treatment uptake and ongoing burden of advanced liver disease complications**

Austhors: Jisoo A. Kwon^1^, Gregory J. Dore^1^, Behzad Hajarizadeh^1^, Maryam Alavi^1^, Heather Valerio^1^, Jason Grebely^1^, Rebecca Guy^1^, Richard T. Gray^1^­

^1^The Kirby Institute, UNSW Sydney, Sydney, New South Wales 2052, Australia

**Supplementary tables**

**S1 Table. Additional model inputs and parameter estimates**

| **Parameters** |  | **Value** | **Reference** |
| --- | --- | --- | --- |
| **Treatment uptake distribution for each disease stage** | F0 | 23-27% | [1, 2] |
|  | F1 | 23-27% |  |
|  | F2 | 15-17% |  |
|  | F3 | 10% |  |
|  | F4 | 20-30% |  |
| **Percentage of people living with HCV who are likely to be diagnosed** | F0 | 64% | Assumption |
|  | F1 | 80% |  |
|  | F2 | 81% |  |
|  | F3 | 85% |  |
|  | F4 | 100% |  |
| **Proportion of people who are under care** | F0 | 100% | Assumption |
|  | F1 | 100% |  |
|  | F2 | 100% |  |
|  | F3 | 100% |  |
|  | F4 | 100% |  |
| **Number of people reeived treatment** | **Year** |  |  |
| **Interferon-based** | 2004  2005  2006  2007  2008  2009  2010  2011  2012  2013  2014  2015 | 1,831  1,847  2,847  3,539  3,664  3,864  3,795  3,053  2,726  3,520  3,643  4,718 |  |
| **DAA** | 2016  2017  2018  2019  2020 | 33,200  20,970  15,210  11,310  8,100 |  |

F0-F4, fibrosis stage 0 to 4; DAA, direct-acting antiviral;

**S2. Table. Years of achieving WHO HCV elimination targets with/without excess alcohol consumption in the model**

| **Years of achieving WHO HCV elimination targets** | | | | |
| --- | --- | --- | --- | --- |
| **With the excess alcohol consumption** | | | | |
|  | **Pessimistic** | **Intermediate** | **Optimistic** |  |
| **90% reduction in new chronic infections** | 2034 | 2032 | 2030 |  |
| **80% of people living with chronic HCV treated** | 2032 | 2030 | 2027 |  |
| **65% reduction in HCV-related mortality** |  |  |  |  |
| **Viraemic only** | 2039 | 2033 | 2023 |  |
| **Viraemic and cured** | >2050 | >2050 | >2050 |  |
| **Without the excess alcohol consumption** | | | | |
|  | **Pessimistic** | **Intermediate** | **Optimistic** |  |
| **90% reduction in new chronic infections** | 2036 | 2034 | 2031 |  |
| **80% of people living with chronic HCV treated** | 2032 | 2030 | 2027 |  |
| **65% reduction in HCV-related mortality** |  |  |  |  |
| **Viraemic only** | 2036 | 2021 | 2021 |  |
| **Viraemic and cured** | >2050 | >2050 | >2050 |  |

**References**

1. Butler T, Simpson M. *National Prison Entrants’ Blood-Borne Virus Survey Report 2004, 2007, 2010, 2013, and 2016*.; 2017.

2. Boelen L, Teutsch S, Wilson DP, et al. Per-event probability of hepatitis C infection during sharing of injecting equipment. *PLoS One*. 2014;9(7):e100749. doi:10.1371/journal.pone.0100749

3. Micallef JM, Kaldor JM, Dore GJ. Spontaneous viral clearance following acute hepatitis C infection: a systematic review of longitudinal studies. *J Viral Hepat*. 2006;13(1):34-41. doi:10.1111/j.1365-2893.2005.00651.x

4. Kwon JA, Anderson J, Kerr CC, et al. Estimating the cost-effectiveness of needle-syringe programs in Australia. *AIDS*. 2012;26(17):2201-2210. doi:10.1097/QAD.0b013e3283578b5d

5. Australian Institute of Criminology. *National Deaths in Custody Program: Deaths in Custody in Australia 2016–17*. Canberra, Australia; 2019.

6. Hallager S, Ladelund S, Christensen PB, et al. Liver-related morbidity and mortality in patients with chronic hepatitis C and cirrhosis with and without sustained virologic response. *Clin Epidemiol*. 2017;9:501-516. doi:10.2147/CLEP.S132072

7. Gane EJ, Stedman CA, Hyland RH, et al. Efficacy of nucleotide polymerase inhibitor sofosbuvir plus the NS5A inhibitor ledipasvir or the NS5B non-nucleoside inhibitor GS-9669 against HCV genotype 1 infection. *Gastroenterology*. 2014;146(3):736-743 e1. doi:10.1053/j.gastro.2013.11.007

8. Lawitz E, Poordad FF, Pang PS, et al. Sofosbuvir and ledipasvir fixed-dose combination with and without ribavirin in treatment-naive and previously treated patients with genotype 1 hepatitis C virus infection (LONESTAR): an open-label, randomised, phase 2 trial. *Lancet*. 2014;383(9916):515-523. doi:10.1016/S0140-6736(13)62121-2

9. Poordad F, Lawitz E, Kowdley K V, et al. Exploratory study of oral combination antiviral therapy for hepatitis C. *N Engl J Med*. 2013;368(1):45-53. doi:10.1056/NEJMoa1208809

10. Feld JJ, Jacobson IM, Hezode C, et al. Sofosbuvir and Velpatasvir for HCV Genotype 1, 2, 4, 5, and 6 Infection. *N Engl J Med*. 2015;373(27):2599-2607. doi:10.1056/NEJMoa1512610

11. Roberts SK, Weltman MD, Crawford DH, et al. Impact of high-dose peginterferon alfa-2A on virological response rates in patients with hepatitis C genotype 1: a randomized controlled trial. *Hepatology*. 2009;50(4):1045-1055. doi:10.1002/hep.23130

12. Hagan LM, Sulkowski MS, Schinazi RF. Cost analysis of sofosbuvir/ribavirin versus sofosbuvir/simeprevir for genotype 1 hepatitis C virus in interferon-ineligible/intolerant individuals. *Hepatology*. 2014;60(1):37-45. doi:10.1002/hep.27151

13. Australian Government: Department of Health. MBS Online: Medicare Benefits Schedule. http://www.mbsonline.gov.au/internet/mbsonline/publishing.nsf/Content/Home. Published 2020.

14. Australian Government: Department of Health. The Pharmaceutical Benefit Scheme. https://www.pbs.gov.au/browse/medicine-listing.

15. Commonwealth of Australia. *National Hospital Cost Data Collection Australian Public Hospitals Cost Report, Round 11*.; 2015.

1. The Kirby Institute. Real world efficacy of antiviral therapy in chronic hepatitis C in Australia (Issue 2). The Kirby Institute, UNSW Sydney; July 2018.

2. The Kirby Institute. Monitoring hepatitis C treatment uptake in Australia (Issue 10). The Kirby Institute, UNSW Sydney; 2019.
